# Factors Associated with Anxiety, Depression, and Stress Levels in High School Students

**DOI:** 10.3390/ejihpe13090129

**Published:** 2023-09-13

**Authors:** Relmu Gedda-Muñoz, Álvaro Fuentez Campos, Alfonso Valenzuela Sakuda, Iván Retamal Torres, Matías Cruz Fuentes, Georgian Badicu, Tomás Herrera-Valenzuela, Pablo Valdés-Badilla

**Affiliations:** 1 Vice-Rectory of Quality Assurance, Universidad Autónoma de Chile, Temuco 4810101, Chile; relmu.gedda@uautonoma.cl; 2 Department of Physical Activity Sciences, Faculty of Education Sciences, Universidad Católica del Maule, Talca 3530000, Chile; alvaro.fuentes@alu.ucm.cl (Á.F.C.); alfonso.valenzuela@alu.ucm.cl (A.V.S.); ivan.retamal@alu.ucm.cl (I.R.T.); matias.cruz@alu.ucm.cl (M.C.F.); 3 Department of Physical Education and Special Motricity, Faculty of Physical Education and Mountain Sports, Transilvania University of Brasov, 500068 Brasov, Romania; 4 Department of Physical Activity, Sports and Health Sciences, Faculty of Medical Sciences, Universidad de Santiago de Chile (USACH), Santiago 8370003, Chile; tomas.herrera@usach.cl; 5 Sports Coach Career, School of Education, Universidad Viña del Mar, Viña del Mar 2520000, Chile

**Keywords:** adolescent psychology, physical activity, educational achievement, students, youth

## Abstract

This study aims to investigate the relationship between anxiety, depression, and stress levels with physical activity level and academic performance in high school students; secondly, this study aims to relate and compare anxiety, depression, and stress levels with physical activity level and academic performance. This is a quantitative, descriptive, and comparative cross-sectional study, which evaluated 443 high school students (48% female; 15.13 ± 1.59 years) belonging to the Maule region, Chile. The Depression, Anxiety, and Stress Questionnaire (DASS-21) and the International Physical Activity Questionnaire (IPAQ) were applied. Academic performance was consulted on language, mathematics, and overall grade point average. The results indicate that vigorous physical activity (OR = 0.504; *p* = 0.017) and high academic performance in mathematics (OR = 0.597; *p* = 0.027) are associated with a reduced risk of depression. In turn, there is a significant inverse correlation between physical activity with anxiety (r = −0.224; *p* = 0.000), depression (r = −0.224; *p* = 0.000) and stress (r = −0.108; *p* = 0.032), while the performance of mathematics is inversely correlated with depression (r = −0.176; *p* = 0.000). On the other hand, significant differences (*p* < 0.05) between anxiety, depression, stress levels, and grade point average were found, with females exhibiting higher scores than males. In conclusion, greater vigorous physical activity and scoring above average in mathematics performance are protective factors against depression.

## 1. Introduction

Adolescence is a crucial stage in the development of individuals, in which different physical, physiological, and psychoemotional changes occur due to the transition from childhood to adulthood [1]. Changes at the psychoemotional level are manifested through variations in behaviour, personality, social relationships, self-perception, and sleep habits, among others, which can increase the risk of developing psychopathological disorders [1,2]. On the other hand, a significant association between higher anxiety and depression and lower academic performance has been reported [3].

According to the World Health Organization [4], depression is a disorder whose main symptoms are sadness, a loss of interest or pleasure, eating disorders, feelings of tiredness, and a lack of concentration. It can also affect academic performance and activities of daily living and, in the worst cases, can lead to suicide [5]. Some factors increase the risk of depression, e.g., a socio-economic situation that puts people at risk of social exclusion, types of interpersonal relationships that diminish self-esteem, the loss of a loved one, and different situations that involve grief processes for parental, familial, or social reasons [6].

High school students spend a significant part of their time at school, making it a space where intense collective and individual experiences converge [2,7]. Specifically, in Chile, high school education is compulsory, and students attend this educational level between the ages of 12 and 18 years. The influence that anxiety, depression, and stress have on academic performance at the school level [3,5,8], including absenteeism [9] and school dropout [10,11], have been widely researched, and their results contribute to generating institutional actions and public policies with the purpose of reducing the impact of these problems. However, school is a dynamic space and must constantly adapt to new scenarios that society and people present it with [12,13]. In Chile, the population aged 12 to 19 years shows a prevalence of moderate to severe depression ranging from 32.6% to 39.8% [14,15]. Previous studies have reported a higher tendency toward depressive symptomatology in females at 24.2%, compared to males at 15.6% [15]. On the other hand, Maykel [16] mentions that academic stress is a general malaise that directly influences physical and emotional aspects in students whose incident factors are internal, such as health conditions, the pressure to develop extracurricular activities, aspirations for the future, among others; external incident factors as also include competitiveness among peers, relationships with teachers, a school environment focused on the results of standardised assessments and a high load of homework or extracurricular academic activities. These aspects affect academic performance and undermine teamwork and self-esteem [16,17,18]. Therefore, the early detection of anxiety symptoms [7], such as depression [6] and stress [2], has been considered of the utmost importance for their timely referral and treatment to reduce the risk of presenting such a disorder in the future or during adulthood.

In addition, students who have regular physical activity practice tend to show greater concentration than those who do not, which improves their cognitive capacity and learning in the academic environment [19]. It has been reported that physical education classes and physical activity are positively related to higher academic performance, especially in mathematical calculations and reading comprehension in schoolchildren [19] and university students [20]. Furthermore, regular physical activity produces favourable effects at the psychoemotional level by reducing anxiety, stress levels, and depressive episodes [21,22]. Therefore, an active lifestyle could become a protective factor against the prevalence of disorders such as anxiety, depression, and stress [23], especially in high school students. In addition to the above, at the national and local levels, there is a lack of information and scientific background to enable relevant and effective political and institutional actions to address these problems. In this sense, the present study has two aims: (i) to investigate the relationship between anxiety, depression and stress levels with the physical activity level and academic performance of high school students and secondly, (ii) to relate and compare anxiety, depression, and stress levels with physical activity level and academic performance.

## 2. Materials and Methods

A descriptive, comparative, cross-sectional, and quantitative study was carried out for this investigation. The Depression, Anxiety, and Stress Questionnaire (DASS-21) [24] and the International Physical Activity Questionnaire (IPAQ) short form [25] were applied. In addition, the student’s academic records were consulted to obtain their academic performance data.

### 2.1. Participants

A sample was selected under an accidental non-probabilistic criterion and consisted of 443 students (48% female: mean age 14.82 ± 1.59; mean height 159.51 ± 6.76 cm; mean weight 57.28 ± 11.02 kg. 52% male: mean age 15.31 ± 1.55; mean height 170.70 ± 9.77 cm; mean weight 66.95 ± 15.52 kg) belonging to different schools in the Maule region, Chile. The inclusion criteria were: (i) aged 12 to 19 years, regardless of sex; (ii) enrolled in a public or private educational institution in the Maule region, Chile; and (iii) being under preventive confinement due to a COVID-19 health alert (voluntary or imposed by health authorities). The exclusion criteria were: (i) not answering the survey in full; (ii) those who had been ill with COVID-19 in the last six months, as this could bias the students’ responses.

All students, together with their parents or tutors, were informed of the scope of the study and accepted informed consent authorizing the use of information for scientific purposes. The study protocol was reviewed and approved by the Scientific Ethics Committee of the Universidad Autónoma de Chile (approval number: N° 18-18; date: 27 December 2018) and was developed following the Declaration of Helsinki.

### 2.2. Anxiety, Depression, and Stress Assessment

Anxiety, depression, and stress were measured through the DASS-21 scale, an instrument with 21 items divided into three dimensions composed of seven statements each, which assessed the levels of depression, anxiety, and stress [24]. Each item had four response alternatives in the Likert scale format scored as 0: Did not apply to me at all; 1: applied to me to some degree, or some of the time; 2: Applied to me to a considerable degree or a good part of the time; 3: Applied to me very much or most of the time.

To answer the questionnaire, the person had to indicate to what extent each item or statement described what he/she felt or what had happened to him/her during the last week. This instrument has the advantage of being a self-report scale, brief, easy to administer and answer, and easy to interpret [24]. Each dimension (depression, anxiety, and stress) reported the following: the higher the value, the greater the degree of symptomatology, with values ranging from 0 to 21 points. The cut-off points per dimension were as follows: (i) Depression: without symptomatology (less than 5 points), mild (5 to 6 points), moderate (7 to 10 points), severe (11 to 13 points), extremely severe (14 to 21 points); (ii) Anxiety: without symptomatology (less than 4 points), mild (4 points), moderate (5 to 7 points), severe (8 to 9 points), extremely severe (10 to 21 points); (iii) Stress: without symptomatology (less than 8 points), mild (8 to 9 points), moderate (10 to 12 points), severe (13 to 16 points), extremely severe (17 to 21 points).

### 2.3. Physical Activity Level

For measurements, the short version of the IPAQ was used, which is composed of seven questions and distributed in four domains: vigorous physical activity, moderate physical activity, low physical activity, and sitting time [25]. The physical activity indicator is expressed both continuously, in MET-minute/week, and categorically, classifying the physical activity level as low, moderate, or high [25]: (i) Vigorous physical activity: 8 MET x minutes x days per week; (ii) Moderate physical activity: 4 MET x minutes x days per week; (iii) Low physical activity: 3.3 MET x minutes x days per week.

### 2.4. Academic Performance

Students’ academic records were consulted considering the subjects that are most predominantly assessed in Chile’s system to measure the quality of education (Sistema de Medición de la Calidad en la Educación, SIMCE for its acronym in Spanish), i.e., language and mathematics. In this sense, the annual averages for the 2022 academic year were obtained for these subjects in addition to the overall grade point average for all students. These data were accurate and accessible indicators to assess academic performance [26], given that they were a reflection of the cognitive development and personal effort of students, as well as being a valuable indicator of the different personal, academic, social, and personal aspects of an individual’s education [27].

### 2.5. Statistical Analysis

The Statistical Package for the Social Sciences (IBM Corp. IBM SPSS Statistics, Version 25.0. Armonk, NY, USA) was used to analyse the data. The variables were subjected to the Kolmogorov–Smirnov test for normality and Levene’s test for the homogeneity of variance, and descriptive analysis was performed, calculating the arithmetic mean and standard deviation. On the other hand, Spearman’s correlation was used to correlate anxiety, depression, and stress with physical activity level and academic performance because these variables did not have a normal distribution. In addition, comparisons by sex were made for anxiety, depression, stress, physical activity level, and academic performance with the Mann–Whitney U test according to the results of the normality test. The effect size (ES) was determined through Cohen’s *d* [28] considering a small (0.20–0.49), moderate (0.50–0.79) or large (≥0.80) effect. Logistic regression analysis was performed to identify the risk factors for DASS-21 dimensions (anxiety, depression, and stress) using the “enter” method, which incorporated the variables of the model in a single step. Odds ratios (OR) and 95% confidence intervals (CI) were used to present the magnitude of association, while the absence of collinearity was verified with a tolerance level of 0.1. Numerical dependent and independent variables were recoded as dummy variables (0 = below average, 1 = above average), with students who scored above average on each variable acting as the reference group. For all cases, a significance level of *p* < 0.05 was established.

## 3. Results

Statistically significant (*p* < 0.05) and inverse correlations were reported for the total sample between anxiety, depression, and stress with vigorous and moderate physical activity (Table 1). Low physical activity showed no significant correlation with the DASS-21 scales, and stress showed significant (*p* < 0.05) and direct correlations with the total sitting time. As for academic performance, depression showed a statistically significant (*p* < 0.05) and inverse correlation with mathematics performance, while grade point average correlated in the same way with depression but not with anxiety or stress. Academic performance in language did not correlate statistically with anxiety, depression, or stress in the total sample. For females, statistically significant (*p* < 0.05) and inverse correlations were observed between depression with moderate and vigorous physical activity, while with low physical activity, the correlation was direct. As for academic performance, anxiety had a statistically significant (*p* < 0.05) and an inverse correlation with mathematics, while depression had a significant (*p* < 0.01) and an inverse correlation with mathematics and the overall grade point average. No significant correlations were reported between stress with physical activity level and academic performance in high school females. On the other hand, there were no statistically significant correlations between anxiety with physical activity level and academic performance in male high school students. Depression correlated significantly (*p* < 0.01) and inversely with academic performance in mathematics, while stress correlated significantly (*p* < 0.05) and directly with the total sitting time.

Table 2 presents the results of anxiety, depression, stress, physical activity level, and academic performance in high school students compared to sex. Statistically significant differences (*p* < 0.01) were found, in which females scored higher levels of anxiety, depression, and stress and had a higher overall grade point average, while males obtained significantly higher values (*p* < 0.01) in vigorous, moderate, low, and total physical activity time, although no differences were found in the total sitting time for sex. Additionally, there were no statistically significant differences in the averages for mathematics and language. In terms of ES analysis, the differences between male and female students concerning vigorous, moderate, and total physical activity, as well as overall academic performance, manifested a small ES. In relation to low physical activity and levels of depression, these differences exhibited a moderate ES. Lastly, the differences in scores for anxiety and stress revealed a large ES.

Table 3 presents the results of the logistic regression for the dichotomous variables of anxiety, depression, and stress regarding the rest of the variables studied. The dimensions for anxiety and stress did not show significant associations with the physical activity and academic performance variables. In the case of the depression dimension, there were statistically significant associations between vigorous physical activity and academic performance in mathematics. Students who engaged in vigorous physical activity were 50.4% more probable (OR = 0.504; *p*-value *=* 0.017) to be below average on the depression subscale, while students with a higher mathematics achievement were 59.7% more probable (OR = 0.597; *p*-value *=* 0.027) to be below the average depression score than students with a lower performance in this area.

## 4. Discussion

The aims of this study were to investigate the relationship between anxiety, depression, and stress levels with the physical activity level and academic performance of high school students; secondly, this study aimed to relate and compare anxiety, depression, and stress levels with physical activity level and academic performance. The main results indicate that there was a statistically significant inverse association between depression and vigorous physical activity, as well as academic performance in mathematics for high school students. This demonstrates that the higher the physical activity level or academic performance, the lower the probability of having a high level of depression. On the other hand, there was a statistically significant inverse correlation between vigorous and moderate physical activity with anxiety, depression, and stress in the total sample, while mathematics performance correlated only with depression. In turn, high school students showed statistically significant differences in anxiety, depression, and stress levels where females scored higher; however, in vigorous, moderate, low, and total physical activity, males showed a higher level than females. Finally, there were significant differences in total academic achievement, where females demonstrated a higher performance than males.

### 4.1. Correlation between Anxiety, Depression and Stress with Physical Activity Level and Academic Performance in High School Students

Regarding the correlations between the study variables, the anxiety, depression, and stress scales maintain an inverse and statistically significant relationship with moderate and vigorous physical activity in the total sample of high school students. This relationship could indicate that the more time spent undertaking moderate or vigorous physical activity, the lower the level of anxiety, depression, and stress reported by students in the DASS-21. In the same vein, when correlating the study variables according to the sex of the students, we found that males did not show a statistically significant relationship between levels of anxiety, depression, and stress with physical activity. On the other hand, females showed a significant and direct correlation between depression and low physical activity and a significant and inverse correlation between depression with moderate and vigorous physical activity. This is consistent with the study by Barrón et al. [23], who found that moderate and vigorous physical activity was associated with a lower prevalence of depression in females. This could be due to the fact that physical activity favours improvements in mood, physical states, and general well-being in individuals, leading to a decrease in symptoms associated with depression [20]. As for the divergent results found in male students, this was because they experienced higher levels of physical activity for all types in this study and lower levels of anxiety, depression, and stress than females. On the other hand, the total sitting time showed a significant and direct relationship with the level of stress in the total sample and for males, with no correlation with anxiety or depression. For female students, there is no correlation between the total sitting time and anxiety, depression, or stress. As for the total time of physical activity, this showed significant and inverse correlations with levels of anxiety and depression, which could demonstrate that the more time spent in physical activity, in general, the lower levels of anxiety and depression an individual has, as previous studies have also demonstrated [29]. Stress, on the other hand, showed no relationship with total physical activity. When differentiating the sample between males and females, total physical activity showed no statistically significant correlation with levels of anxiety, depression, or stress.

In academic terms, anxiety did not show a statistically significant relationship with academic performance for mathematics, language, and grade points average for males in the total sample. In the case of females, anxiety did show a significant and inverse correlation with performance in mathematics. Regarding depression, it surfaced as the dimension exhibiting the highest correlation with academic performance. It manifested a significant and inverse relationship with academic performance in mathematics across the total sample, encompassing both male and female students. Furthermore, a statistically significant inverse correlation was revealed between the overall grade point average within the total sample and among female students. This shows that better performance in mathematics or higher grade point averages can lead to a lower occurrence of elevated levels of depression [3,30]. In this respect, better academic performance is a combination of various aspects and factors. While aspects external to students are highly influential, such as the material conditions of schools, the pedagogical skills of teachers, the social and cultural environment from which students come, and their family’s social, cultural, and economic capital [31], it is also true that there are relevant individual elements. Among the internal or individual elements that are linked to academic performance, these include having a higher academic self-concept and self-esteem [17,26], higher physical activity and healthy eating habits [22,32], and developing study habits [8], among others. Thus, it is possible to link better academic performance with lower depression levels. For its part, stress did not present statistically significant correlations with academic performance in the total sample, nor when divided by sex, while the only academic performance variable that did not present significant relationships with the DASS-21 scale was academic performance in language. Overall, the results obtained are similar to those reported in previous studies [3,30], which indicate that students with better academic performance have a lower prevalence of anxiety, depression, and stress.

### 4.2. Sex Comparison of Anxiety, Depression, Stress, Physical Activity Level and Academic Performance in High School Students

Concerning the comparison by sex of the high school students analysed, it is possible to confirm some of the findings evidenced in the correlations. Females had significantly higher scores on the anxiety, depression, and stress scales than males. This coincides with other studies [15,33,34], which indicate a higher prevalence of anxiety, depression, and stress in females when compared to males. This is a complex and relevant fact, considering that during the high school stage, students are in their adolescence, and these symptoms increase as they become older, mainly due to the physical, hormonal, and social changes to which they are exposed [33]. Complementing this background, adolescent females may feel greater social pressure to fit social and physical stereotypes compared to males and show lower levels of self-esteem [7,33,35]. However, the differences in anxiety, depression, and stress levels between female and male high school students are not only attributable to the changes experienced during adolescence [7] but rather a wider phenomenon, encompassing social expectations, cultural influences, gender roles, and social inequalities, in which families and schools need to be involved, and governments need to develop plans and policies to promote better levels of psychoemotional health in adolescents.

In terms of physical activity, males are more likely to engage in low, moderate, vigorous, and total physical activity than females of the same educational level. According to the scientific literature, males tend to feel more attracted to improving their physical fitness; they participate in sporting competitions [36] and find, more frequently than females, an incentive in sports practice [20]. This could allow us to understand the differences found in the present study. Additionally, it has been reported in studies with high school students that more physically active males have a lower prevalence of anxiety, depression, and stress [7,20]. On the other hand, the total sitting times did not show significant differences between both groups, which is similar to findings provided by previous studies in Chilean students [29]. The difference found between male and female students could be linked to sex stereotypes that are strongly rooted in our societies, as well as in schools, where hegemonic attributes of masculinity are related to physical aspects and performances during adolescence [37,38].

Academic performance in mathematics and language did not show statistically significant differences when comparing students by sex. In the case of academic performance measured as the overall grade point average, there were significant differences, where females exhibited higher grades than males. This is consistent with previous studies [7,8,39], which show that at the school level, females have better academic performance in subjects such as language, mathematics, and grade points average than males. Although several studies agree on this point, it should not be normalised that at the high school level, females perform better academically than males and, at the same time, show higher levels of anxiety, depression, and stress [7]. In addition, as previously indicated, at the high school level, females have a lower level of self-esteem than males [7,35], which creates a worrying picture for the schools, families, and public agencies responsible for education.

### 4.3. Association between Anxiety, Depression and Stress with Physical Activity Level and Academic Performance in High School Students

Referring to the risk factors associated with increased levels of anxiety, depression, and stress, it was identified that both physical activity and academic performance in mathematics influenced depression. Specifically, it was found that more vigorous physical activity in high school students decreased the probability of high levels of depression by 50.4%, while higher mathematics performance decreased the probability of depression by 59.7%. This is similar to what has been found in previous studies, which shows the protective role of physical activity against psychoemotional pathologies [22], highlighting the importance of encouraging and favouring regular physical activity practice in high school students, as well as the need for schools to promote prevention programmes for physical and emotional health in adolescents. Concerning academic performance in mathematics, it is important to highlight that when a student achieves high grades, it is due to a combination of various factors [8,17,22,26,31,32]. The cultivation of their aptitude for specific subjects since childhood, the material provisions offered by their family or school, the presence of motivating teachers proficient in pedagogical strategies that facilitate significant learning, a stimulating learning environment, and supportive families are among these factors. In essence, when we assert that maths grades serve as protective factors against depression, it is imperative to grasp that grades represent an outcome contingent upon foundational conditions that facilitate the achievement of these outcomes. Moreover, the study results are consistent with previous studies showing a significant association between healthy habits, academic self-concept, and better academic performance with lower levels of anxiety, depression, and stress in high school students [3,26,30]. These findings relate to the fact that students who maintain better academic performance also have greater protective factors for their emotional health. However, this is not necessarily associated with adolescents’ self-management capacity or individual capabilities. Instead, it is associated with the material and symbolic conditions related to learning, which in various studies have been associated with the social and family background of high school students [10,31] rather than with adolescents’ innate aptitudes. While the present study did not inquire into students’ socioeconomic conditions, its findings allow us to provide evidence against a contingent phenomenon for our society, post-COVID-19 pandemic, where the mental health of adolescents has been impaired [17,35]; it requires joint work from families, schools and public policies, to strengthen learning processes, promote positive recognition experiences within schools [40], as well as improve physical and emotional health.

### 4.4. Study Limitations and Strengths

Among the possible limitations of this study are the following: (i) the cross-sectional design did not allow for causal relationships; (ii) the collection of data through voluntary online surveys involved the self-reporting of physical activity, anxiety, depression, and stress levels based on student recall, in addition to accidental non-probabilistic sampling, resulting in analyses that were correlational and associative rather than based on causal inference; (iii) during data collection, no information was gathered regarding students diagnosed with emotional or psychological pathologies, or the use of medication that could affect their emotional state; and (iv) it was necessary to consider that the correlations presented between the DASS-21 and the levels of physical activity and academic performance, although statistically significant, presented relatively low *r* values, showing that the strength of these linear relationships is limited. The main study strengths include the sample size (*n* = 443) and the collection of data in different educational establishments, in addition to the use of instruments validated by the Chilean educational system. These facts can allow us to expand the use of this instrument to different educational contexts.

### 4.5. Practical Implications and Future Studies Directions

Based on the findings of the present study, moderate and vigorous physical activity practice, as well as higher performance in mathematics, are protective factors against depression. This is relevant, as depression is one of the main causes of school absenteeism and dropout [9,12]: a trend that has increased during the COVID-19 pandemic [10]. In this sense, recent studies support the early detection of depression in order to achieve more effective treatments and more inclusive environments within educational settings [11,13,16]. Therefore, it is advisable to periodically assess psychoemotional variables, which can be accomplished by easy-to-use instruments such as those used in our study. In this sense, future studies could be developed in two directions: (i) expanding and deepening the utilisation of the DASS-21 questionnaire in the school context, aiming to monitor anxiety, depression, and stress levels in high school students, as well as uncovering new insights according to variables such as the socioeconomic level of families, parents’ level of education, area of residence (urban or rural), among others; and (ii) longitudinal studies that incorporate objective data on students’ physical activity and clinical diagnoses in relation to emotional well-being can be advanced, enabling cases to be tracked throughout the school’s trajectory until the end of high school. This endeavour would provide enhanced evidence to support institutional initiatives and inform public policies.

## 5. Conclusions

Depression is associated with physical activity level and academic performance in high school students, and, specifically, higher levels of vigorous physical activity and achieving above-average performance in mathematics grades are protective factors against depression. On the other hand, there is a statistically significant inverse correlation between anxiety, depression, and stress with physical activity (vigorous and moderate) levels, as well as between performance in mathematics and levels of anxiety and depression. Finally, high school females show significantly higher levels of anxiety, depression, stress, and grade points average compared to males at the same educational level, while males show significantly higher physical activity levels compared with females.

## Figures and Tables

**Table 1:** Correlation between anxiety, depression and stress with physical activity level and academic performance among high school students.

Group	Variables	Anxiety		Depression		Stress	
		*r* Value	*p*-Value	*r* Value	*p*-Value	*r* Value	*p*-Value
Total sample (*n* = 443)	Vigorous PA	−0.224	0.000 **	−0.201	0.000 **	−0.108	0.032 *
	Moderate PA	−0.178	0.000 **	−0.127	0.011 *	−0.119	0.018 *
	Low PA	0.003	0.946	0.072	0.154	0.024	0.632
	Sitting time	0.070	0.163	−0.028	0.581	0.122	0.015 *
	Total PA	−0.124	0.013 *	−0.103	0.040 *	−0.035	0.490
	Mathematics	−0.088	0.068	−0.176	0.000 **	−0.093	0.054
	Language	0.015	0.755	−0.018	0.703	0.033	0.491
	Overall GPA	−0.010	0.827	−0.097	0.042 *	0.002	0.960
Females (*n* = 213)	Vigorous PA	−0.074	0.316	−0.166	0.023 *	−0.064	0.383
	Moderate PA	−0.110	0.134	−0.165	0.024 *	−0.077	0.292
	Low PA	0.108	0.140	0.176	0.016 *	0.095	0.195
	Sitting time	0.052	0.478	0.001	0.986	0.090	0.223
	Total PA	−0.032	0.665	−0.094	0.201	−0.003	0.969
	Mathematics	−0.146	0.046 *	−0.203	0.005 **	−0.124	0.090
	Language	0.005	0.942	−0.079	0.285	0.021	0.770
	Overall GPA	−0.135	0.065	−0.188	0.010 **	−0.087	0.235
Males (*n* = 230)	Vigorous PA	−0.044	0.531	−0.033	0.637	0.119	0.085
	Moderate PA	−0.005	0.947	0.034	0.628	0.014	0.842
	Low PA	0.077	0.264	0.062	0.372	0.057	0.408
	Sitting time	0.093	0.178	0.064	0.358	0.171	0.013 *
	Total AF	0.050	0.472	0.010	0.882	0.126	0.068
	Mathematics	−0.050	0.469	−0.194	0.005 **	−0.079	0.255
	Language	−0.034	0.622	−0.045	0.516	−0.004	0.949
	Overall GPA	−0.013	0.853	−0.119	0.086	0.010	0.882

PA: Physical activity. GPA: Grades point average. *r*: Spearman correlation. *p*: significance. *: *p* < 0.05. **: *p* < 0.01.

**Table 2:** Comparison of anxiety, depression, stress, physical activity level and academic performance among high school students by sex.

Total Sample (*n* = 443)	Males (*n* = 230)		Females (*n* = 213)		*p*-Value	ES
	Mean	SD	Mean	SD		
Anxiety (score)	4.75	4.60	10.74	5.54	0.000	1.38 ^†^
Depression (score)	7.0	5.27	11.04	5.61	0.000	0.74 ^°^
Stress (score)	6.82	5.36	11.48	5.20	0.000	1.15 ^†^
Vigorous PA (min)	62.90	49.91	43.37	46.64	0.000	0.32 ^¶^
Moderate PA (min)	53.28	48.38	41.81	40.59	0.000	0.25 ^¶^
Low PA (min)	69.38	142.62	40.46	48.16	0.004	0.057 ^°^
Sitting time (min)	197.40	196.35	209.81	238.30	0.742	0.095 ^°^
Total PA (MET/min/week)	4130.51	5142.28	2036.47	2323.36	0.000	0.33 ^¶^
Mathematics (grades average)	6.01	0.82	6.01	0.76	0.860	0.00
Language (grades average)	5.83	0.71	5.91	0.73	0.214	0.00
Overall GPA	5.98	0.67	6.10	0.63	0.034	0.32 ^¶^

SD: Standard deviation. PA: physical activity. Min: minutes. Grades average: 1.0 to 7.0 according to the Chilean school system. GPA: grades point average. *p*-value: statistical significance according to Mann–Whitney U. ES: Effect size. ^¶^ = small effect. ^°^ = moderate effect. ^†^ = large effect.

**Table 3:** Factors associated with anxiety, depression, and stress levels in high school students.

Total Sample (*n* = 443)	Anxiety				Depression				Stress			
	OR	CI 95%		*p*-Value	OR	CI 95%		*p*-Value	OR	CI 95%		*p*-Value
Above the average of Vigorous PA	0.619	0.354	1.080	0.091	**0.504**	**0.287**	**0.886**	**0.017**	1.007	0.583	1.740	0.980
Above the average of Moderate PA	0.723	0.425	1.230	0.232	1.010	0.594	1.716	0.971	0.744	0.440	1.257	0.269
Above the average of Low PA	1.400	0.802	2.446	0.237	0.943	0.540	1.646	0.835	1.018	0.589	1.759	0.950
Above the average of Sitting time	1.171	0.771	1.779	0.458	1.158	0.761	1.763	0.494	1.342	0.888	2.029	0.163
Above the average of Total PA	1.119	0.597	2.096	0.726	1.396	0.744	2.618	0.299	1.299	0.703	2.404	0.404
Above the average Mathematics	0.689	0.436	1.087	0.109	**0.597**	**0.378**	**0.943**	**0.027**	0.717	0.456	1.128	0.150
Above the average Language	1.258	0.789	2.004	0.335	1.305	0.816	2.086	0.266	1.281	0.808	2.029	0.292
Above the average Overall GPA	1.104	0.700	1.740	0.671	0.740	0.470	1.167	0.196	1.050	0.669	1.646	0.832

PA: physical activity. GPA: grades point average. OR: odds Ratio, where a value > 1 indicates a higher probability of being below average on the anxiety, depression, and stress subscales. CI: confidence intervals. *p*-value: significance, in bold *p* < 0.05.

## Data Availability

The datasets generated and/or analysed during the current research are available from the corresponding author on reasonable request.
